# Predicting ischemic stroke patients’ prognosis changes using machine learning in a nationwide stroke registry

**DOI:** 10.1007/s11517-024-03073-4

**Published:** 2024-04-05

**Authors:** Ching-Heng Lin, Yi-An Chen, Jiann-Shing Jeng, Yu Sun, Cheng-Yu Wei, Po-Yen Yeh, Wei-Lun Chang, Yang C. Fann, Kai-Cheng Hsu, Jiunn-Tay Lee, Taiwan Stroke Registry Investigators

**Affiliations:** 1 Division of Intramural Research, Disorders and Stroke, National Institute of Neurological, National Institutes of Health, 9000 Rockville Pike, Bethesda, MD 20892 USA; 2 Center for Artificial Intelligence in Medicine, Chang Gung Memorial Hospital, Taoyuan, Taiwan; 3 Bachelor Program in Artificial Intelligence, Chang Gung University, Taoyuan, Taiwan; 4 Stroke Center and Department of Neurology, National Taiwan University Hospital, Taipei, Taiwan; 5 Department of Neurology, En Chu Kong Hospital, New Taipei City, Taiwan; 6 Department of Exercise and Health Promotion, College of Kinesiology and Health, Chinese Culture University, Taipei, Taiwan; 7 Department of Neurology, St. Martin de Porres Hospital, Chiayi, Taiwan; 8 Department of Neurology, Show Chwan Memorial Hospital, Changhua County, Taiwan; 9 Department of Medicine, China Medical University, Taichung, Taiwan; 10 Artificial Intelligence Center for Medical Diagnosis, China Medical University Hospital, No. 2, Yude Rd., North Dist., Taichung, 404332 Taiwan; 11 Department of Neurology, China Medical University Hospital, Taichung, Taiwan; 12 Department of Neurology, Tri-Service General Hospital, National Defense Medical Center, Taipei, Taiwan Republic of China

**Keywords:** Ischemic stroke, Machine learning, Prognosis changes, Risk factors, Nation-wide registry database

## Abstract

Accurately predicting the prognosis of ischemic stroke patients after discharge is crucial for physicians to plan for long-term health care. Although previous studies have demonstrated that machine learning (ML) shows reasonably accurate stroke outcome predictions with limited datasets, to identify specific clinical features associated with prognosis changes after stroke that could aid physicians and patients in devising improved recovery care plans have been challenging. This study aimed to overcome these gaps by utilizing a large national stroke registry database to assess various prediction models that estimate how patients’ prognosis changes over time with associated clinical factors. To properly evaluate the best predictive approaches currently available and avoid prejudice, this study employed three different prognosis prediction models including a statistical logistic regression model, commonly used clinical-based scores, and a latest high-performance ML-based XGBoost model. The study revealed that the XGBoost model outperformed other two traditional models, achieving an AUROC of 0.929 in predicting the prognosis changes of stroke patients followed for 3 months. In addition, the XGBoost model maintained remarkably high precision even when using only selected 20 most relevant clinical features compared to full clinical datasets used in the study. These selected features closely correlated with significant changes in clinical outcomes for stroke patients and showed to be effective for predicting prognosis changes after discharge, allowing physicians to make optimal decisions regarding their patients’ recovery.

**Graphical Abstract:**

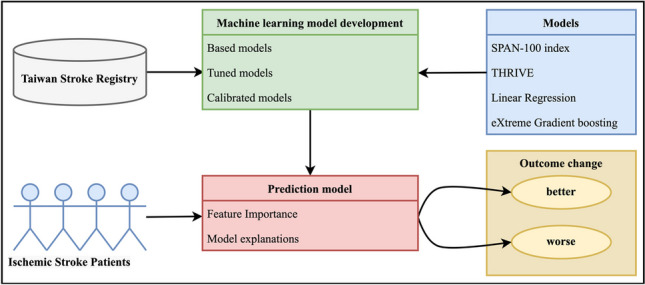

**Supplementary Information:**

The online version contains supplementary material available at 10.1007/s11517-024-03073-4.

## Introduction

Stroke is the second leading cause of mortality in the global population and is also a significant burden within the global health community [1]. Many patients are unable to promptly receive necessary treatments, leading to post-discharge disability. This critically impacts their quality of life with excessive burdens on both their families and society.

Recent studies have demonstrated that machine learning (ML) is a powerful tool for predicting stroke functional outcomes [2–4]. For instance, one study explored various ML models to predict outcomes for patients in a national stroke registry with their best model achieved an area under the receiver operating characteristic curve (AUROC) of 0.94 for both ischemic and hemorrhagic stroke patients using admission assessment, treatment, and inpatient data. The study also found a strong association between the prediction results and the future recovery and health conditions of the stroke patients [3]. Fernandez-Lozano et al. utilized a random forest-based outcome prediction model to forecast the prognosis of ischemic stroke patients with pre-hospital and hospitalization data, along with 2-day follow-up data, to project the patients’ 30-day prognosis outcomes. The study achieved AUROC values of 0.90 and 0.75 for predicting patients’ mortality and morbidity, respectively [5].

Recently, Monteiro et al. utilized logistic regression (LR), decision tree (DT), support vector machine (SVM), and extreme gradient boosting (XGboost) models to predict the modified Rankin Scale (mRS) at the 3-month follow-up. The ML approach outperformed models based on clinical scores, such as the Acute Stroke Registry and Analysis of Lausanne (ASTRAL) [6]. The superb performance of the XGBoost model has attracted many ML researchers, such as latest studies reported by Chen, et al. [7], Price, et al. [8], and again Moore, et al. [9] that further demonstrated the XGBoost model for its exceptional performance in various clinical cohorts and studies in terms of both speed and accuracy for clinical applications.

Although previous ML-based studies have demonstrated a reasonable level of accuracy in predicting stroke outcomes, they have not identified any associated clinical features that could assist physicians and patients in developing improved recovery care plans. In addition, a recent review conducted by Wang et al. [10] found that many ML-based studies on stroke outcome prediction utilized small sample populations only ranging from 70 to 3184 patients. These studies also considered a limited number of clinical features, ranging from 4 to 152, respectively, which may produce biased predictions and conclusions. As far as our knowledge extends, no studies have been reported that employed prediction models specifically focusing on analyzing clinical factors that may influence the prognosis of ischemic stroke patients within a large cohort, with particular emphasis on changes in functional outcomes after discharge which is crucial to the stroke recovery and toward the development of clinical applications.

In this study, we aimed to establish a predictive model that accurately predicts patient’s prognosis changes and identifies associated key clinical factors that may potentially impact changes in subsequent functional outcomes following patient discharge. To achieve this, we utilized the Taiwan Stroke Registry (TSR), a large nationwide multicenter stroke registry database, with a substantial number of data variables [11]. to investigate various prognosis prediction models with associated clinical factors affecting prognoses of ischemic stroke patients at 1-month and 3-month follow-ups after discharge. To avoid prejudice in conducting a comprehensive evaluation of different predictive models based on changes in functional outcomes with associated clinical features, we examined and utilized both traditional and the latest ML-based approaches for comparison. These approaches included the latest proven high-performance machine learning-based XGboost model, a traditional statistical model based on logistic regression [12], as well as commonly used clinical score-based models that incorporate patient age and the NIH Stroke Scale Index (a.k.a. SPAN-100 index) [13], and the totaled health risks in vascular events (THRIVE) [14]. Both SPAN-100 index and THRIVE are commonly used clinical feature-based scores by physicians and health researcher to associate and predict clinical response, outcome, mortality, and risk of in ischemic stroke patients.

## Key characteristics of functional outcome changes of ischemic stroke patients after discharge in the TSR database

**Fig. 1:**
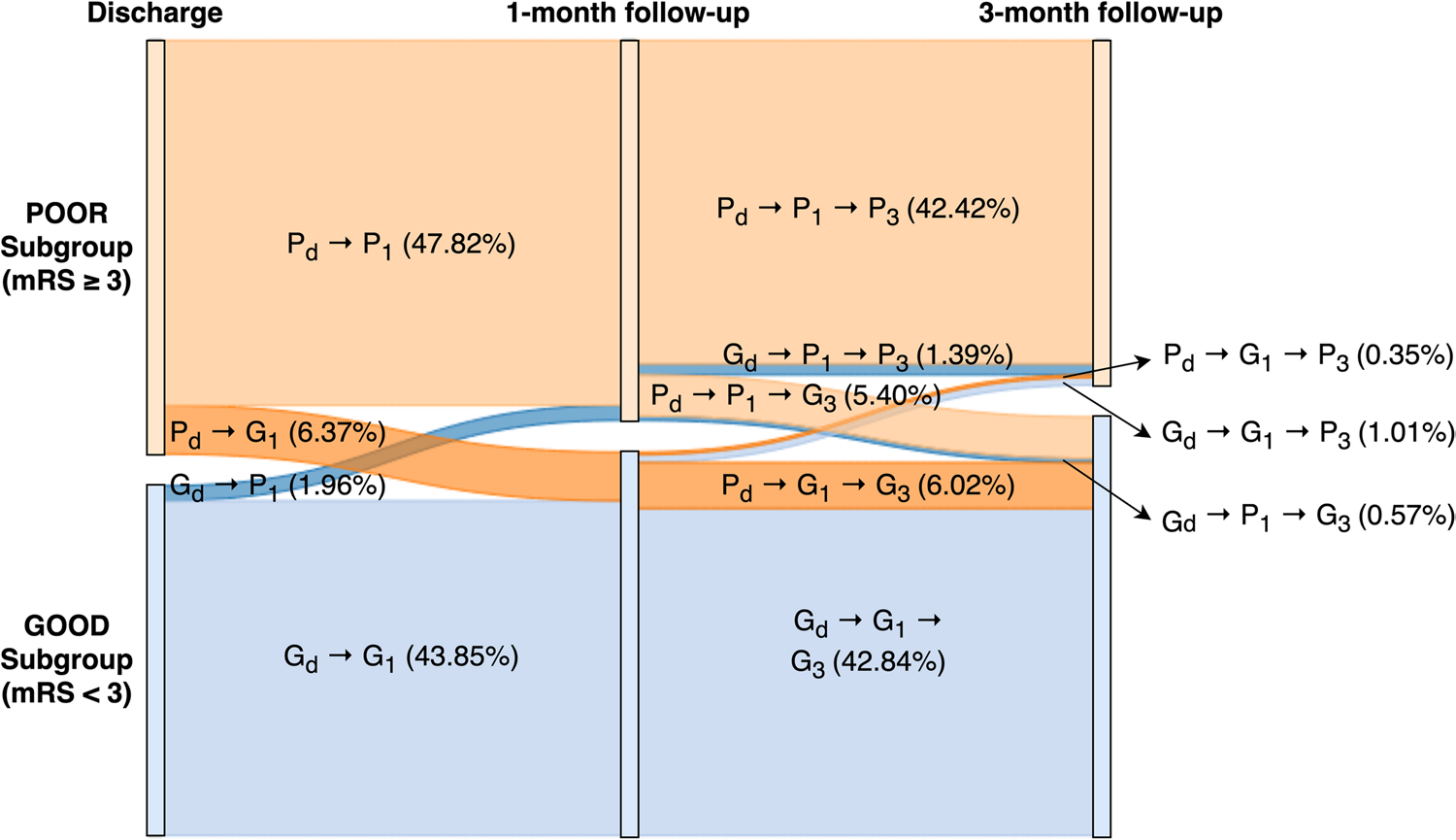
A flow diagram depicting the functional outcome changes of ischemic stroke patients (Poor (P) vs Good (G)) from discharge (d), 1-month (1 m), to 3-month (3 m) follow-ups. For instances, P_d_ indicates the functional outcome of the group of individuals was poor at discharge; P_1m_ indicates the functional outcome of the group of individuals was poor at 1-month follow-up; P_3m_ indicates the functional outcome of the group of individuals was poor at 3-month follow-up; G_d_ indicates the functional outcome of the group of individuals was good at discharge; G_1m_ indicates the functional outcome of the group of individuals was good at 1-month follow-up; G_3m_ indicates the functional outcome of the group of individuals was good at 3-month follow-up. The width of the bands is proportional to the flow rate, which represents the percentage of patients in each subgroup that went from discharge to 1-month follow-up and again into 3-month follow-up. For example, P_d_ → P_1m_ (47.82%) indicates 47.82% of study patients remained poor outcome from discharge to 1 month, and P_d_ → P_1m_ → G_3m_ (5.40%) indicates among those 47.82% of patients 5.40% of them improved from poor outcomes to good outcomes

To explore and illustrate the trend of ischemic stroke patient’s functional outcome measures, a flow diagram of TSR patients was created from over 60,000 ischemic stroke patients’ modified Rankin Scale (mRS) at discharge, 1-month and 3-month follow-ups from 2006 to 2020. The diagram is shown in Fig. 1. In this study, the good outcome was defined as mRS score < 3 and the poor outcome as mRS score ≥ 3 as commonly accepted by the stroke research community [15]. Figure 1 demonstrated that a majority of patients remained at the same outcome status during the entire observation period (up to 3 months), while only 8.33% of total cases exhibited a change in their functional outcome at 1-month, and 7.33% of cases changed at 3-month follow-up. In addition, four distinct types of outcome changes were noticed from discharge to 1-month follow-up, with different alterations of outcome changes were observed between 1- and 3-month follow-ups. For example, in Fig. 1, the dark blue color described fewer cases transitioning from good to poor outcome from discharge to 1-month follow-up (denoted as G_d_ → P_1m_) with most cases remaining at a poor outcome status between 1- and 3-month follow-up (G_d_ → P_1m_ → P_3m_), however, with about 30% of cases improved back to a better health condition at 3-month follow-up (G_d_ → P_1m_ → G_3m_). Moreover, there were significant poor outcome cases (6.37% of total studied population) at discharge that turned into good outcome (P_d_ → G_1m_) at 1-month follow-up with the majority of cases remaining at the same health condition. This is the first time a detailed flow diagram was presented that clearly demonstrated the case distributions of outcome changes from discharge to 3-month follow-up from a large population-based stroke registry. The objective of this study was to identify the best prediction model for the outcome change prognosis at 1-month and 3-month follow-ups with associated clinical features that may promote good outcome changes for stroke patients after discharge. The study also aimed to identify clinical risk factors that may trigger poor or promote good outcome changes at long-term follow-ups. The findings of this study will help physicians better understand and plan for their patient recovery and health care management after stroke.

## Materials and methods

### Ethical approval

The TSR database encrypts personal information of the patients to protect privacy and provides researchers with anonymous identification numbers associated with relevant claims information, including sex, date of birth, medical services received, and prescriptions. This study was approved to fulfill the condition for exemption by the Institutional Review Board (IRB) of China Medical University, Taichung, Taiwan (CMUH102-REC1-086(CR-7) that waived the consent requirement. All investigations employed in this study were performed in accordance with the relevant clinical research guidelines and regulations.

### Patient population and cohort selections for the study

**Fig. 2:**
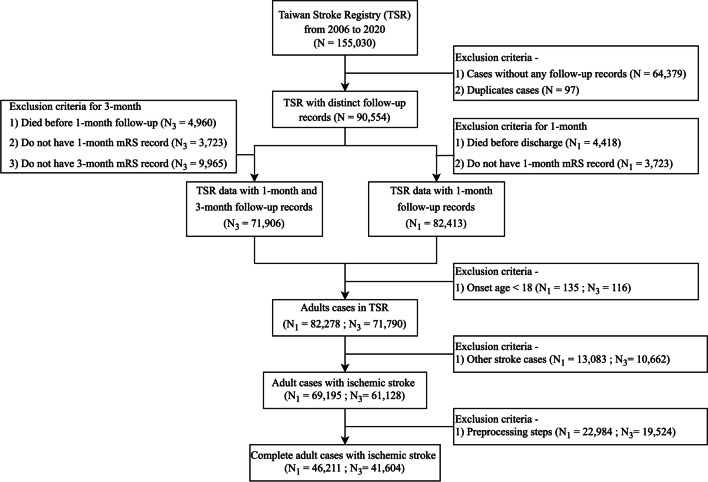
A flow chart diagram illustrating patient cohort selection criteria from Taiwan Stroke Registry (TSR) for the study. mRS, modified Rankin Scale; N_1_, the number of records in the 1-month follow-up dataset. N_3_, the number of records in the 3-month follow-up dataset

The datasets supporting the investigation and findings of this study are available upon requests from the corresponding authors. For the development and evaluation of predictive models, 155,030 stroke patient records from TSR with 531 clinical variables recorded between 2006 and 2020 were retrospectively selected. TSR mainly collected stroke patients’ admission and assessment data, clinical assessments during hospitalization, functional outcome measurements, in-hospital complications, medical history, laboratory test results, electrocardiography, computed tomography findings, magnetic resonance imaging (MRI) findings, medications during admission, functional outcome measures, discharge status, and follow-up information for retrospective research use. As presented in Fig. 2, to select represented high quality datasets for this study, 64,379 cases without any available follow-up information and 97 duplicated records were processed and removed. The remaining 90,554 records were employed for the further screening of 1- and 3-month cohort sets. For the 1-month cohort dataset, 82,413 cases were kept by removing 4418 patients who died before discharge and 3723 patients without a 1-month follow-up mRS record. In addition, 135 patients less than 18 years old, and 13,083 with other identified stroke diagnosis (e.g., hemorrhage stroke) were removed, leaving 69,195 adult patients being selected for 1-month follow-up cohort. For the 3-month cohort dataset, similar selection and extraction steps were performed to delete those lack of 1- and 3-month mRS records that left with 3723 and 9965 cases. After dropping 116 non-adult cases and 10,662 cases diagnosed as other strokes, 61,128 ischemic stroke patients were retained as the 3-month follow-up dataset. Those collected adult cases with ischemic stroke would then be applied with the data preprocessing for missing values and amputation steps (see Supplementary Fig. S1). After data preprocessing steps were performed, two high quality 1- and 3-month follow-up ischemic stroke cohorts with 46,198 and 41,604 subjects, respectively, were used in the study.

### Definitions of the prediction targets

The prediction target of this study was to determine whether each case’s 1- and 3-month follow-up mRS conditions (good or poor outcome) were changed from that of at discharge. The study divided patients into two condition subgroups, good and poor outcomes at discharge. For the good outcome (mRS < 3) at discharge group, the patient’s health conditions could remain unchanged or become deteriorated (i.e., poor outcome) at 1-month follow-up. Similarly, the patients in poor outcome (mRS ≥ 3) at discharge group could remain as unchanged or improved. Similarly, the 3-month outcome groups followed the same definitions as those of at 1-month follow-up. The prediction models were trained to predict changes in two condition groups’ functional outcomes and validated for such models’ predictive power at 1- and 3-month, respectively. The good outcome change from discharge to 1-month follow-up dataset was denoted as Gd → 1 m and the poor outcome change from discharge to 1-month follow-up dataset was denoted as Pd → 1 m. Similarly, the good outcome change from discharge to 3-month follow-up dataset was denoted as Gd → 3 m and the poor outcome change from discharge to 3-month follow-up dataset was denoted as Pd → 3 m.

### Predictive models and cross validation

**Fig. 3:**
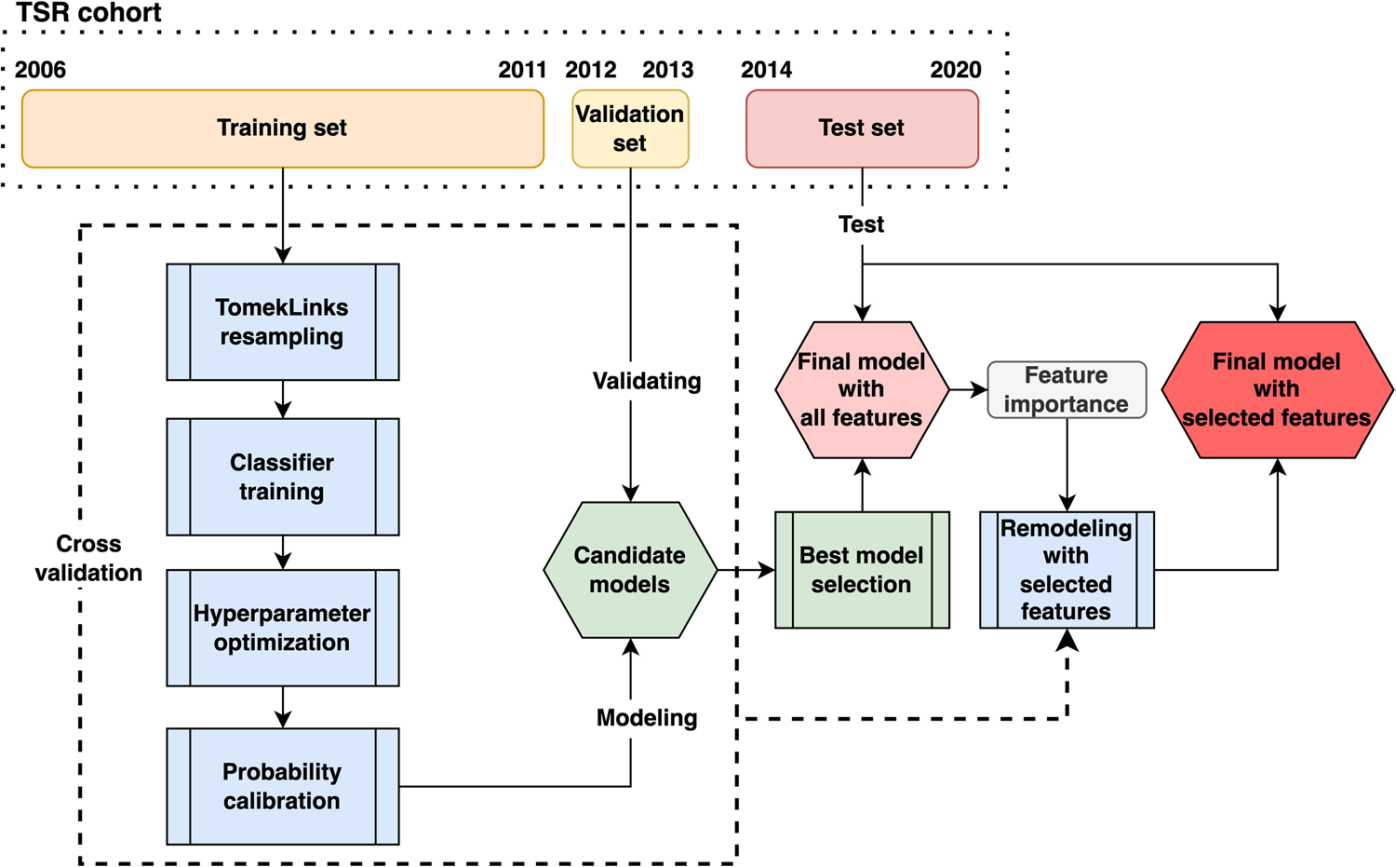
Model building steps and cross validation processes for stroke prognosis change predictions

The predictive model building steps with cross validation processes are demonstrated in Fig. 3. Based on the admission years, the TSR cohort data sets were designed and split into three sets: training (2006–2011), validation (2012–2013), and test (2014–2020) datasets. The cross-validation based on time series splits mimics and simulates the “real world” clinical forecasting environment, that is, using prior years’ populations to predict future stroke patient’s outcome changes. Training and validation datasets were used in each cross-validation round for model evaluation and feature selections. As shown in Fig. 1, the distributions of outcome changes were largely unbalanced; thus, Tomek Links[16, 17], a known under-sampling method, was applied on majority distributions to tackle this challenge before building the prediction models. For model fine tuning steps, hyperparameter optimization with randomized search [18] and probability calibration with isotonic regression [19] were applied. In each cross-validation round, individual model was evaluated by the validation dataset for the best model selection.

To compare and validate predictability obtained from each built model, this study employed two well-validated and simple-to-use clinical outcome prediction tools, the SPAN-100 index and the THRIVE score, for ischemic stroke with test datasets as the baseline performance [20–23]. The SPAN-100 index is the sum of age (in years) and admission National Institutes of Health Stroke Scale (NIHSS) (0–42) with a decision split point of 100. The THRIVE score employs a 0 to 9 point scale that consists of age, admission NIHSS, and medical history such as hypertension (HT), diabetes mellitus (DM), and atrial fibrillation (AF). The THRIVE total score ranges from 0 to 9 with some previously reported studies split the score into three groups (0–2, 3–5, and 6–9) [14, 20, 24–27], and others split the score into two groups (0–5 or 6–9) [28]. This study split the THRIVE score into two subgroups by two different cutoff points to examine good or poor outcome subgroups based on mRS [29]. One was THRIVE-3 (whether greater or equal to three) and the other was THRIVE-6 (whether greater or equal to six) for subsequent analyses. Taking the THRIVE-6 as an example, the predicted outcome is poor when the THRIVE is no less than 6, while patients with THRIVE score less than 6 are anticipated to have good outcome at 1- and 3-month follow-ups. Subsequently, according to the definition of the prediction targets, 1- and 3-month outcome’s labels were converted into unchanged and changed (either improved or deteriorated) classes for model evaluations and comparisons.

For the statistical analysis model, differences within two cohort (1-month and 3-month) sets’ training, validation, and test datasets were investigated by standardized mean difference (SMD). In this study, an SMD of 0 indicates no inconsistencies among the subsets, whereas that of over 0.2, 0.5, and 0.8 represent small, medium, and large disparity, respectively [30]. Model performance was evaluated using well-accepted AUROC, specificity, sensitivity, and positive predictive value (PPV). If the predictions were not derived from probabilities, the AUROC was acquired from a confusion matrix which is $\frac{1}{2}\left[\left(\frac{{\text{tp}}}{{\text{tp}}+{\text{fn}}}\right)+\left(\frac{{\text{tn}}}{{\text{tn}}+{\text{fp}}}\right)\right]$; Specificity is $\frac{{\text{tn}}}{{\text{tn}}+{\text{fp}}}$. Sensitivity is $\frac{{\text{tp}}}{{\text{tp}}+{\text{fn}}}$; PPV is $\frac{{\text{Sensitivity}}\times \mathrm{ Prevalence}}{{\text{Sensitivity}}*{\text{Specificity}}+\left(1-{\text{Specificity}}\right)\times (1-{\text{Prevalence}})}$ where ${\text{prevalence}}= \frac{{\text{tp}}+{\text{fn}}}{{\text{tp}}+{\text{fp}}+{\text{fn}}+{\text{tn}}}$[31]. Note that tp was a case that truly changed the status, fp was a case that not truly changed the status, fn was a case that not truly remained the status, and tn was a case that truly remained the status. Statistical analysis was performed with R tableone package (version 0.12.0) and Python sklearn package (version 0.24.2).

Feature selection is a crucial step in identifying a small subset of input features that can still achieve reasonable predictive performance. Reducing the number of required feature inputs makes prediction models more practical since it is often difficult to collect hundreds of input features during stroke triage without missing or making errors in real-world clinical practices. In the XGboost model used in this study, feature importance was computed from Gini impurity: $1- \sum_{i=1}^{j}{p}_{i}^{2}$, where $j$ is the number of classes, ${p}_{i}$ is the proportion of samples labeled with class *i*. All features were ranked in descending order by their importance. Two types of thresholds were adopted to select the most important features. One was to extract a specific number of the top influential features, while the other was to establish ${{\text{threshold}}}_{{\text{min}}}={\text{minimum}}\left(\upsigma \right)+\mathrm{standard deviation}\left(\sigma \right)$[32] where $\sigma$ is the list of feature importance in which the zeros were removed. The selected features were further used to re-train a pruned XGboost model (see Fig. 3).

## Results

### Patient population characteristics

The study included 46,198 and 41,604 ischemic stroke patients from TSR with respective 1-month and 3-month follow-up information as described in the “Materials and methods”. These two cohorts were further divided into training, validation, and test datasets for further investigations. The summary of patient characteristics of three datasets from two cohorts based on their case numbers, age, sex, NIHSS, and mRS as well as their corresponding outcomes is shown in Table 1 with no significant variations across datasets. However, the authors did notice subtle but important observations in each cohort and individual dataset that may provide additional insights on the prediction results. For example, male population is about 60% of ischemic stroke cases in TSR with mean age of about 69. The mean mRS at 1-month follow-up was found about 0.13 – 0.18 scale lower than that at discharge (i.e., improvement) versus 0.27 – 0.34 lower at 3-month follow-up. These findings indicate overall positive trends with improvement of outcomes 90 days after discharge. The same positive trends were reflected in the numbers of cases with good outcome changes at 3-month follow-up (e.g., more than 50% of patient population in each dataset).

**Table 1:** Summary of patient characteristics and clinical outcomes for three datasets from two cohorts

		Training dataset	Validation dataset	Test dataset	SMD
1-month cohort set	Case number, *n*	27,553	6502	12,143	-
	Male, *n* (%)	16,146 (59)	3963 (61)	7484 (62)	0.041
	Age, (mean ± SD) yr	67.77 ± 12.21	68.95 ± 12.70	69.31 ± 12.92	0.081
	Admission NIHSS (mean ± SD)	5.79 ± 6.32	6.92 ± 6.75	6.98 ± 7.00	0.120
	Discharge NIHSS (mean ± SD)	4.79 ± 5.65	5.17 ± 6.10	5.44 ± 6.41	0.072
	Discharged mRS (mean ± SD)	2.60 ± 1.46	2.75 ± 1.44	2.77 ± 1.50	0.078
	1-month mRS (mean ± SD)	2.42 ± 1.62	2.62 ± 1.61	2.63 ± 1.66	0.089
	1-month good outcome, *n* (%)	14,880 (54)	3144 (48)	5949 (49)	0.075
3-month cohort set	Case number, *n*	25,465	5429	10,710	-
	Male, *n* (%)	14,911 (59)	3330 (61)	6617 (62)	0.044
	Age, (mean ± SD) yr	67.68 ± 12.16	68.85 ± 12.61	69.15 ± 12.89	0.078
	Admission NIHSS (mean ± SD)	5.69 ± 6.21	6.64 ± 6.55	6.66 ± 6.69	0.101
	Discharge NIHSS (mean ± SD)	4.66 ± 5.48	4.80 ± 5.76	5.08 ± 6.00	0.049
	Discharged mRS (mean ± SD)	2.57 ± 1.46	2.72 ± 1.42	2.71 ± 1.48	0.065
	1-month mRS (mean ± SD)	2.23 ± 1.79	2.43 ± 1.76	2.44 ± 1.78	0.078
	3-month good outcome, *n* (%)	15,288 (60)	2974 (55)	5877 (55)	0.071

*n*, the exact case number; *yr*, age in year; *SD*, standard deviation; *SMD*, standardized mean difference; *NIHSS*, National Institutes of Health Stroke Scale; *mRS*, modified Rankin Scale. *good outcome*, mRS < 3

### Comparison of the models for the prediction of prognosis changes

As shown in Table 2, the AUROCs of clinical scores model, statistical analysis model, and ML-based XGboost model were obtained from two different cohort datasets with two discharge outcome conditions (i.e., good vs poor outcomes). The ML-based XGboost model significantly outperformed both statistical and clinical score-based models. The ML model’s performance had higher predictability in poor outcome subgroups of 1- and 3-month follow-ups (e.g., Pd → 1 m and Pd → 3 m) than those with good outcome changes at discharge subgroups (e.g., Gd → 1 m and Gd → 3 m). In addition, the AUROCs of both logistic regression and XGboost models obtained from the 3-month cohort datasets were found 8–10% better than those of the 1-month cohort datasets. For the clinical score-based model, all AUROCs were merely around 0.5 that is, with nearly no predictability [33]. These results indicated that there were more important clinical features in the clinical score model other than age, NIHSS, HT, DM, or AF combined that contributed to the outcome changes in ischemic stroke patients. Compared to the clinical score model, statistical (logistic regression) and XGboost models outperformed with AUROCs by approximately 20 to 35%, some with AUROCs over 0.9, which implies its high predictably in outcome prognosis. Between the two models, XGboost provided better performance and predictability, which the authors chose to further investigate clinical features relevant to prognosis changes that could be utilized in future clinical applications. The details of each model’s performance other than AUROC, including their sensitivities, specificities, and PPVs, were provided in Supplementary Table S1, S2, and S3 for references.

**Table 2:** The area under the receiver operating characteristic (AUROC) curves of three predictive models investigated

Model approaches	Model	Inputs	1-month cohort set		3-month cohort set	
			G_d→1 m_	P_d→1 m_	G_d→3 m_	P_d→3 m_
Clinical scores	SPAN-100	Age and admitted NIHSS	0.500	0.571	0.505	0.570
	THRIVE-3	Age, admitted NIHSS, hypertension, diabetes mellitus, and atrial fibrillation	0.565	0.620	0.548	0.606
	THRIVE-6	Age, admitted NIHSS, hypertension, diabetes mellitus, and atrial fibrillation	0.497	0.561	0.500	0.559
Statistical Analysis	Logistic regression	TSR-collected variables	0.708	0.829	0.826	0.918
Machine learning	XGboost	TSR-collected variables	**0.727**	**0.841**	**0.857**	**0.929**

*SPAN-100*, stroke prognostication using age and NIH stroke scale index; *THRIVE-3*, totaled health risks in vascular events with cutoff point being 3; *THRIVE-6*, totaled health risks in vascular events with cutoff point being 6; *XGboost*, extreme gradient boosting; *NIHSS*, National Institutes of Health Stroke Scale; *TSR*, Taiwan Stroke Registry; *G*_*d→1 m*_, good outcome change from discharge to 1-month follow-up; *P*_*d→1 m*_, poor outcome at discharge to 1-month follow-up; *G*_*d→3 m*_, good outcome change from discharge to 3-month follow-up; *P*_*d→3 m*_, poor outcome change from discharge to 3-month follow-up

### Predictive performance with selected features based on the XGboost model

Given that XGboost was the best prediction model, we further explored its predictive performance for selecting a small subset of clinical features at different thresholds that could still maintain its reasonable predictability (i.e., similar AUROCs) compared to that with all clinical features employed. This allowed them to elucidate and assess crucial clinical features that physicians can focus on during the stroke triage while maintaining their confidence in the model predictability. Supplementary Fig. S2 demonstrates the AUROCs of four outcome change subgroups at discharge from two cohorts (1-month vs 3-month) with numbers of selected features ranked by their importance obtained by the XGboost model. The total number of all features used for the 1-month cohort data set was 228, while those of the 3-month cohort data set was 229. The number of clinical features selected at the threshold_min_ for Gd → 1 m, Pd → 1 m, Gd → 3 m, and Pd → 3 m subgroups were 6, 8, 8, and 3 respectively. The AUROCs did not change significantly when top 20 features were selected for the prediction model compared to those with all features used. All the AUROCs using the XGboost model with the threshold_min_ predictors (i.e., crucial clinical features) showed satisfying predictability from all four subgroup datasets except for the Gd → 1 m. The selected threshold_min_ predictors were mostly from the Barthel index assessments which indicated patients’ mobility and functional independence such as transfering and bathing were highly associated with prognosis changes after stroke. In addition, those patients with large artery atherosclerosis, previous cerebrovascular accident, and hypertriglyceridemia history were likely to show improvement over time as evidenced in their good 3-month outcome changes. More selected features with detailed information such as ranking of feature importance and the ROC curves are available in Supplementary Table S4 and Figures S3, S4, S5, and S6).

## Discussion

The study focused on predicting changes in functional outcomes at two key follow-up time points with associated key clinical features instead of directly predicting their mRS outcome scores. This approach allowed for better clinical applications to assist physicians caring for ischemic stroke patients after discharge. As presented in the study results, the XGboost and LR models were clearly better discriminators than the clinical scores-based model. Between the two, XGboost showed the best performance to investigate key clinical features associated with the prognosis changes. Compared to the clinical scores model with only a few variables collected at admission, the XGboost model employed clinical variables obtained at different time points throughout the triage ranging from admission, assessments, treatment to discharge, and follow-up outcomes. Similar observations and performance were reported by M. Monteiro et al. [6] who also verified that great AUROC could be achieved to some extent as long as features at various time points were added to the analysis.

**Fig. 4:**
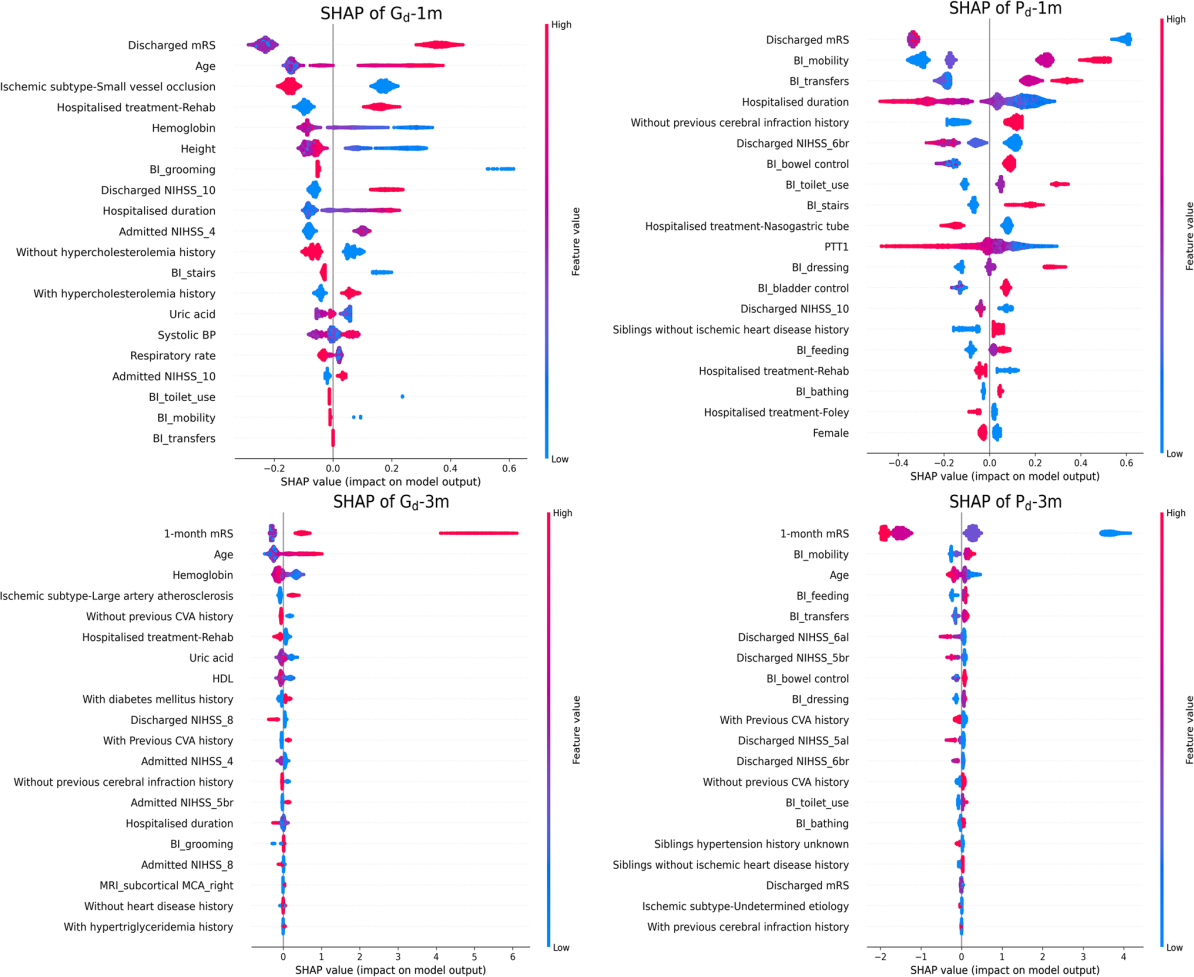
The SHAP diagrams that explained whether each selected predictor would cause the follow-up outcomes to change or remain the same in each evaluated subgroup. A is the SHAP diagram of G_d→_1 m. B is the SHAP diagram of P_d→_1 m. C is the SHAP diagram of G_d→_3 m. D is the SHAP diagram of P_d→_3 m. G_d→1 m_, Good outcome change from discharge to 1-month follow-up; P_d→1 m_, poor outcome change from discharge to 1-month follow-up; G_d→3 m_, good outcome change from discharge to 3-month follow-up; P_d→3 m_, poor outcome change from discharge to 3-month follow-up; mRS, the modified Rankin Scale; BI, the Barthel Index; NIHSS, the National Institutes of Health Stroke Scale; BP, blood pressure; PTT1, the first test of partial thromboplastin time; MRI, magnetic resonance imaging; MCA, middle cerebral artery; CVA, cerebrovascular accident; HDL, high-density lipoprotein

The study further employed SHapley Additive exPlanations (SHAP) algorithm [34, 35] to elucidate and assess XGboost selected clinical features or risk factors that caused positive and/or negative prognosis changes. SHAP is a unified framework that helps explain the origins of predictions by assigning each predictor (in this case clinical feature) an impact value to a specific prediction significance (i.e., SHAP value). In this study, only the top 20 predictors of each cohort group were selected and shown in summary plots of SHAP value obtained (Fig. 4). The summary plots of four prognosis outcome change subgroups (i.e., Gd → 1 m, Pd → 1 m, Gd → 3 m, and Pd → 3 m) were presented with SHAP value (SV) and feature value (FV). When the SV is greater than 0 (positive), it implies that the follow-up mRS would change; on the other hand, SV less than 0 signifies that the follow-up mRS would remain consistent or unchanged. In this study, the FV is presented as a blue to red gradient with blue, purple, and red representing low, middle, and high FV, respectively, relative to their degrees of importance. Taking age in each follow-up subgroup as an example, the model indicated that older age patients were more likely to alter their outcome status from good to poor in both Gd-1 m and Gd-3 m subgroups but remained in poor outcomes status in both Pd → 1 m and Pd → 3 m subgroups. That is, older age was found to be associated with a high probability of having poor prognosis at the follow-ups regardless of their discharged mRS in TSR population.

This study clearly demonstrated that the presented ML-based XGboost model can be utilized to predict the long-term outcome changes after ischemic stroke. Rather than directly predicting the outcome score, stroke care physicians can now use patients’ outcomes at discharge with our prediction model to evaluate their follow-up mRS changes for a personalized recovery plan. In addition, from the summary plots of SHAP values obtained from 1- and 3-month follow-up subgroups, these important risk factors associated with each prognosis change can be used to better triage patients following each outcome change subgroup. It is important to note that our predictive model should be used in conjunction with physician’s clinical knowledge and judgment, but not as a substitute.

Many studies have previously shown the high predictability of stroke functional outcome at discharge using various ML models. However, as shown in our study with a large and diverse population, most patients were found to have the same outcome status during the entire observation period (i.e., up to 3-month follow-up); thus, functional outcome alone could not be predicted reliably. In this study, we demonstrated the changes in functional outcomes after discharge as a better prognosis indicator and compared their predictabilities and performance with different computational approaches (e.g., clinical score-based model, statistical LR model, vs XGboost ML model). We utilized the best prediction model to ascertain clinical features crucial to the recovery and care management after stroke based on a large nationwide multi-center stroke registry database. Compared with clinical scores or simple statistical LR models, our predictive model based on XGboost ML algorithm had significantly higher performance in predicting ischemic stroke patient’s outcome changes. In addition, selected few key clinical features such as age, functional independence, and mobility were shown to have profound impacts on the recovery of ischemic stroke patients.

In summary, this study allows physicians to use the predicted results in their clinical practices for planning an optimal personalized care plan aiming for improved recovery. Future studies will focus on building an effective prediction tool to help clinicians select an optimal treatment plan with positive prognosis changes for stroke patients and to prevent any potential deterioration in their future outcomes after discharge. This study has several limitations that should be noted. The majority of the TSRs used in this study were from Asian patients; thus, the model’s performance for other populations is uncertain. Patients with missing data may have recovered to a point where follow-up is deemed unnecessary or could have been relocated to a nursing home due to poor functional status, rendering them unable to attend follow-up appointments.

### Supplementary Information

Below is the link to the electronic supplementary material.

Supplementary file1 (DOCX 2011 KB)
