# Supplementary material for: Predicting ischemic stroke patients’ prognosis changes using machine learning in a nationwide stroke registry
Source: Med Biol Eng Comput. 2024 Apr 5;62(8):2343–54. doi: 10.1007/s11517-024-03073-4 (PMC11289005; doi:10.1007/s11517-024-03073-4)
Supplement: Supplementary file 1 — Supplementary file1 (DOCX 2011 KB) [file 11517_2024_3073_MOESM1_ESM.docx]

**Data Preprocessing**

The data preprocessing in the study explained how we partitioned ischemic stroke cases into training, validation, and test sets. The former section discussed the change of feature size, while the latter focused on the alteration of study population. In the beginning, we removed irrelevant and timestamp variables such as metadata and 6-month follow-up information, so the number of independent variables halved from 531 to 280 to 254. Within 254 variables, we classified them into categorical and continuous groups and amputated the abnormal figures. For the categorical group, anomalous values which were not in the codebook were amputated. For the continuous group, several steps were executed; the placeholders and negative figures for non-negative features were amputated, and the values exceeding the 1.5 interquartile range (IQR) were also amputated. After amputating abnormal values, we removed any clinical features with more than 40% missingness; the total number of variables thus decreased from 254 to 228. We fitted the standard scaler to standardize features by removing the mean and scaling to unit variance on the training set and then used the scaler to transform the data on the training, validation, and test sets. The next step was to tidy discharged modified Rankin Score (mRS), Barthel Index (BI), and National Institutes of Health Stroke Scale (NIHSS) scores, and those values would be amputated according to the following descriptions.

1. The conflicts amid mRS, BI, and NIHSS, e.g.,
   1. All values of those features being 0
   2. Discharged BI and mRS being 0
   3. Discharged BI and NIHSS being 0
2. A row having any missingness
3. Utilizing locally estimated scatterplot smoothing (LOESS) algorithm to detect outliers of discharged mRS

The records whose categorical features had any amputated values or missingness were eliminated from 69,195 down to 46,211 (1-month cohort set) and from 61,128 down to 41,614 (3-month cohortstan set). Based on the admission year, we hereafter split both cohort data sets into their own training (2006 - 2011), validation (2012 - 2013), and test (2014 - 2020) sets. Multiple Imputation by Chained Equations (MICE) library (version 3.13.0) in R was implemented to impute the missingness of the continuous features. We set the number of imputations and iterations of MICE as 5 (multiple imputation) and 20, respectively, and we assigned the imputation method as predictive mean matching (pmm), which is the most commonly used technique for continuous variables. The training set was first input to the MICE to find the patterns and associations amid all variables, and an imputation model was thus developed. We then fed the validation and test data sets to the established imputation model to infer which value each missingness should be based on the training set’s imputation rules described above.


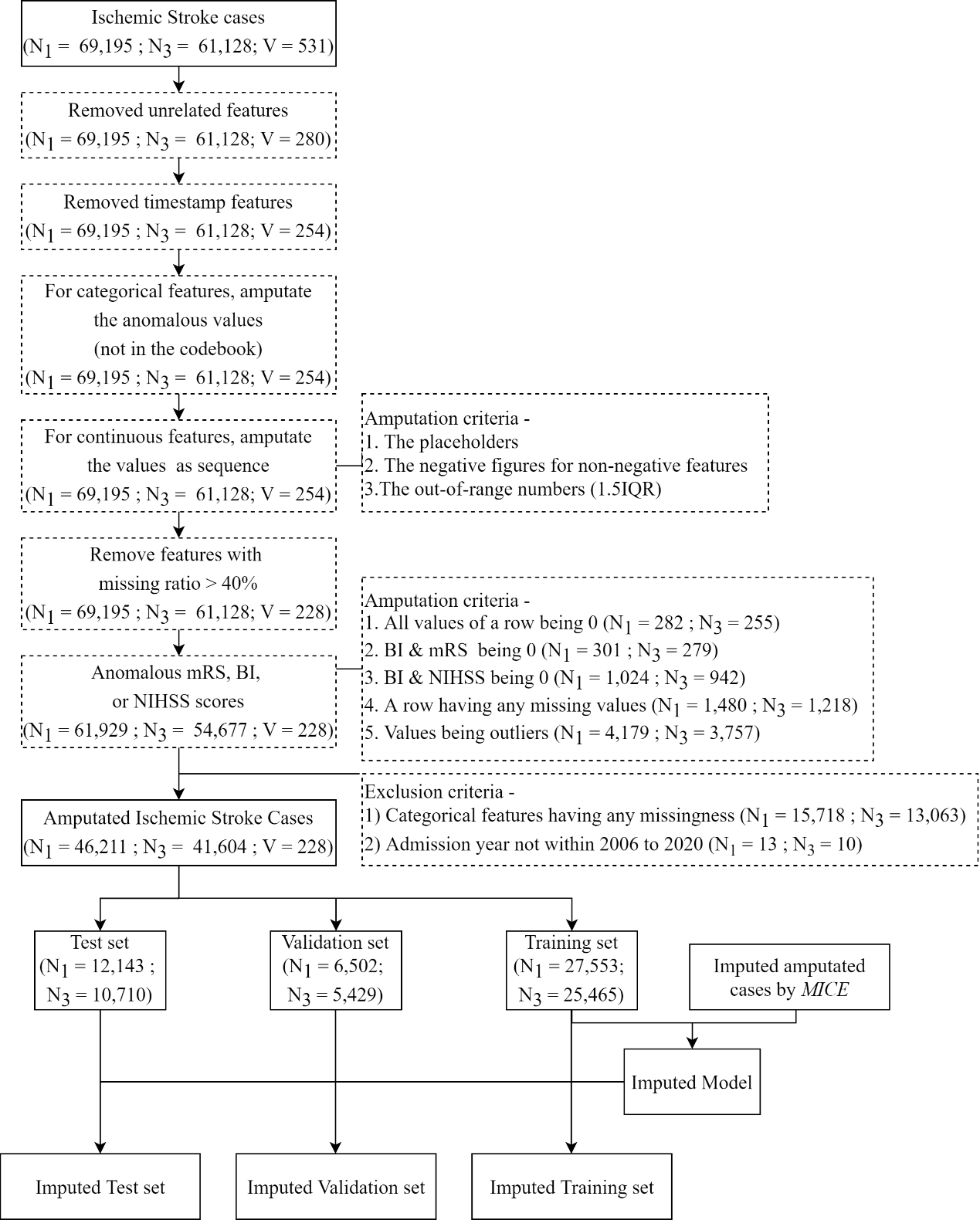


**Fig.S1.** A flow chart illustrating the data preprocessing that split ischemic stroke cases into training, validation, and test data set. N1: the number of records in 1-month follow-up dataset; N3: the number of records in 3-month follow-up dataset; V: the number of variables; IQR: the interquartile range; mRS: the modified Rankin Scale; BI: the Barthel Index; NIHSS: the National Institutes of Health Stroke Scale; MICE: the Multiple Imputation by Chained Equations; Training set: the ischemic cases from 2006 to 2011; Validation set: the ischemic cases from 2012 to 2013; Test set: the ischemic cases from 2014 to 2020.

**Performances other than AUROC**

**Table S1.** The sensitivity of predictive models.

| **Approach** | | **Model** | **1-month cohort set** | | **3-month cohort set** | |
| --- | --- | --- | --- | --- | --- | --- |
|  |  |  | G_d_-1m | P_d_-1m | G_d_-3m | P_d_-3m |
| Clinical score | SPAN-100 | | 0.005 | 0.988 | 0.015 | 0.985 |
|  | THRIVE-3 | | 0.430 | 0.605 | 0.396 | 0.571 |
|  | THRIVE-6 | | 0.000 | 0.979 | 0.005 | 0.979 |
| Statistic model | Logistic regression | | 0.886 | 0.849 | 0.649 | 0.868 |
| Machine learning | XGboost – threshold_min_ * | | 0.746 | 0.837 | 0.624 | 0.957 |
|  | XGboost – 20 | | 0.637 | 0.847 | 0.728 | 0.838 |
|  | XGboost – all* | | 0.813 | 0.859 | 0.644 | 0.844 |

G_d_-1m: good outcome at discharge to 1-month follow-up; P_d_-1m: poor outcome at discharge to 1-month follow-up; G_d_-3m: good outcome at discharge to 3-month follow-up; P_d_-3m: poor outcome at discharge to 3-month follow-up; SPAN-100: stroke prognostication using age and NIH stroke scale index; THRIVE-3: totaled health risks in vascular events with cut-off point being 3; THRIVE-6: totaled health risks in vascular events with cut-off point being 6; XGboost: extreme gradient boosting; threshold_min_ *: the number of threshold_min_ of G_d_-1m, P_d_-1m, G_d_-3m, and P_d_-3m were 6, 8, 8, and 3, respectively; all*: the 1-month cohort set possessed 228 features, while the 3-month cohort set possessed 229 features.

**Table S2.** The specificity of predictive models.

| **Approach** | | **Model** | **1-month cohort set** | | **3-month cohort set** | |
| --- | --- | --- | --- | --- | --- | --- |
|  |  |  | G_d_-1m | P_d_-1m | G_d_-3m | P_d_-3m |
| Clinical score | SPAN-100 | | 0.996 | 0.154 | 0.996 | 0.155 |
|  | THRIVE-3 | | 0.699 | 0.636 | 0.699 | 0.641 |
|  | THRIVE-6 | | 0.994 | 0.142 | 0.994 | 0.139 |
| Statistic model | Logistic regression | | 0.991 | 0.974 | 0.983 | 0.960 |
| Machine learning | XGboost – threshold_min_ * | | 0.983 | 0.972 | 0.983 | 0.985 |
|  | XGboost – 20 | | 0.982 | 0.974 | 0.986 | 0.954 |
|  | XGboost – all* | | 0.987 | 0.976 | 0.984 | 0.955 |

G_d_-1m: good outcome at discharge to 1-month follow-up; P_d_-1m: poor outcome at discharge to 1-month follow-up; G_d_-3m: good outcome at discharge to 3-month follow-up; P_d_-3m: poor outcome at discharge to 3-month follow-up; SPAN-100: stroke prognostication using age and NIH stroke scale index; THRIVE-3: totaled health risks in vascular events with cut-off point being 3; THRIVE-6: totaled health risks in vascular events with cut-off point being 6; XGboost: extreme gradient boosting; threshold_min_ *: the number of threshold_min_ of G_d_-1m, P_d_-1m, G_d_-3m, and P_d_-3m were 6, 8, 8, and 3, respectively; all*: the 1-month cohort set possessed 228 features, while the 3-month cohort set possessed 229 features.

**Table S3.** The positive predictive value of predictive models.

| **Approach** | | **Model** | **1-month cohort set** | | **3-month cohort set** | |
| --- | --- | --- | --- | --- | --- | --- |
|  |  |  | G_d_-1m | P_d_-1m | G_d_-3m | P_d_-3m |
| Clinical score | SPAN-100 | | 0.042 | 0.123 | 0.136 | 0.226 |
|  | THRIVE-3 | | 0.050 | 0.167 | 0.053 | 0.285 |
|  | THRIVE-6 | | 0.000 | 0.121 | 0.036 | 0.221 |
| Statistic model | Logistic regression | | 0.779 | 0.798 | 0.620 | 0.844 |
| Machine learning | XGboost – threshold_min_ * | | 0.624 | 0.785 | 0.611 | 0.940 |
|  | XGboost – 20 | | 0.560 | 0.796 | 0.694 | 0.820 |
|  | XGboost – all* | | 0.698 | 0.811 | 0.630 | 0.825 |

G_d_-1m: good outcome at discharge to 1-month follow-up; P_d_-1m: poor outcome at discharge to 1-month follow-up; G_d_-3m: good outcome at discharge to 3-month follow-up; P_d_-3m: poor outcome at discharge to 3-month follow-up; SPAN-100: stroke prognostication using age and NIH stroke scale index; THRIVE-3: totaled health risks in vascular events with cut-off point being 3; THRIVE-6: totaled health risks in vascular events with cut-off point being 6; XGboost: extreme gradient boosting; threshold_min_ *: the number of threshold_min_ of G_d_-1m, P_d_-1m, G_d_-3m, and P_d_-3m were 6, 8, 8, and 3, respectively; all*: the 1-month cohort set possessed 228 features, while the 3-month cohort set possessed 229 features.

**List of selected features**


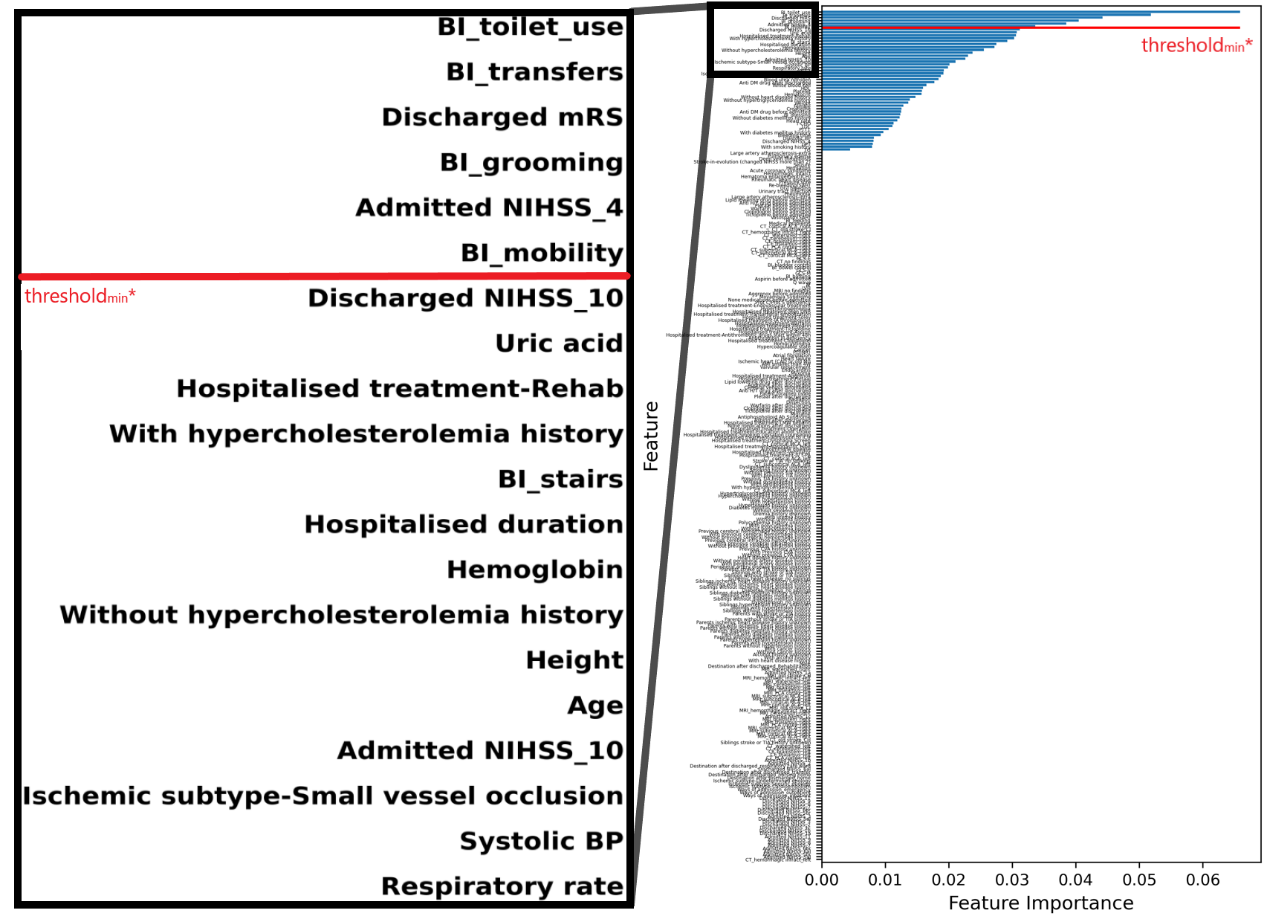


**Fig.S2.** The top 20 selected features from XGboost algorithm, which analyzed patients from good outcome at discharge to the 1-month follow-up. threshold_min_*: the number of threshold_min_’s features from good outcome at discharge to the 1-month follow-up was 6.


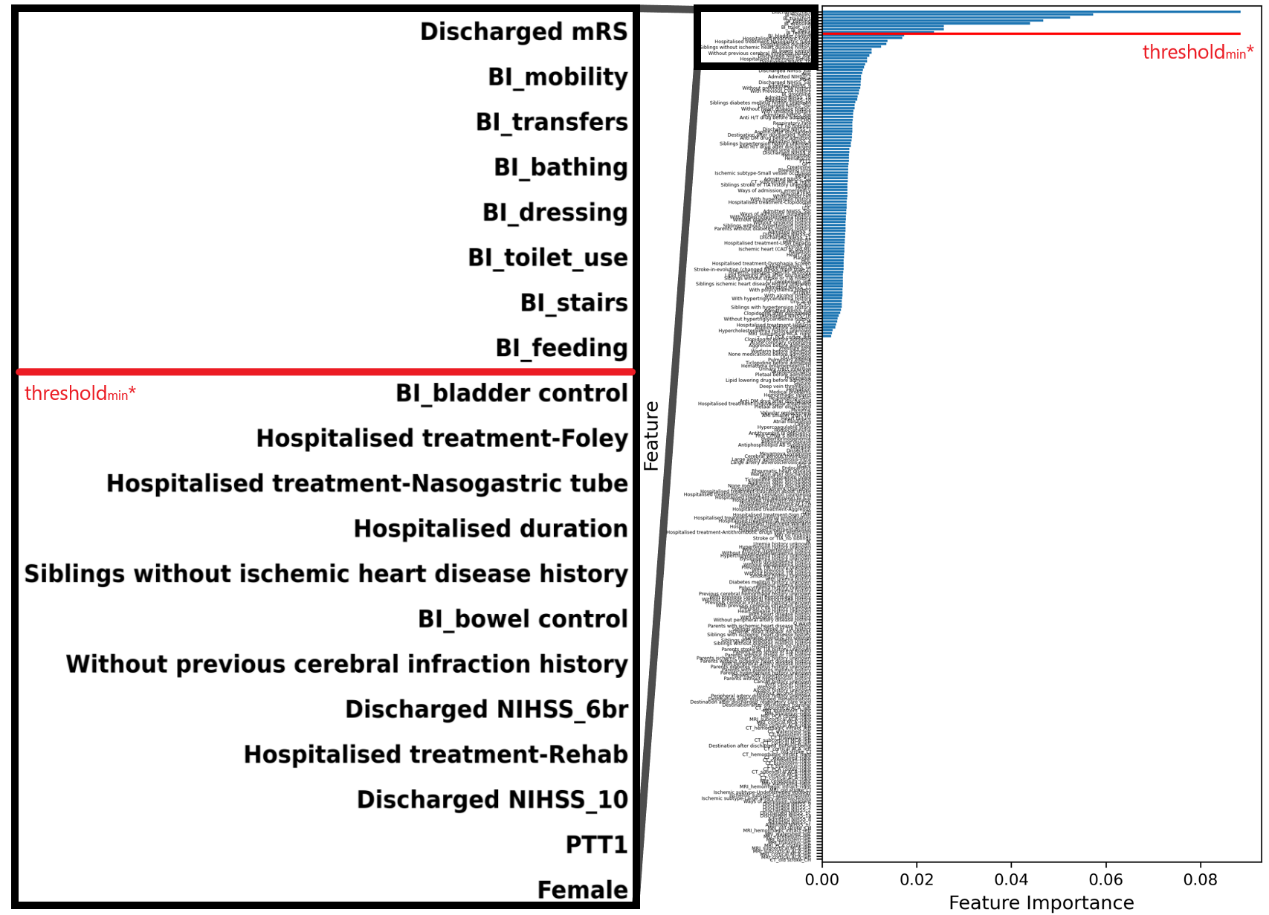


**Fig.S3.** The top 20 selected features from XGboost algorithm, which analyzed patients from poor outcome at discharge to the 1-month follow-up. threshold_min_*: the number of threshold_min_’s features from poor outcome at discharge to the 1-month follow-up was 8.


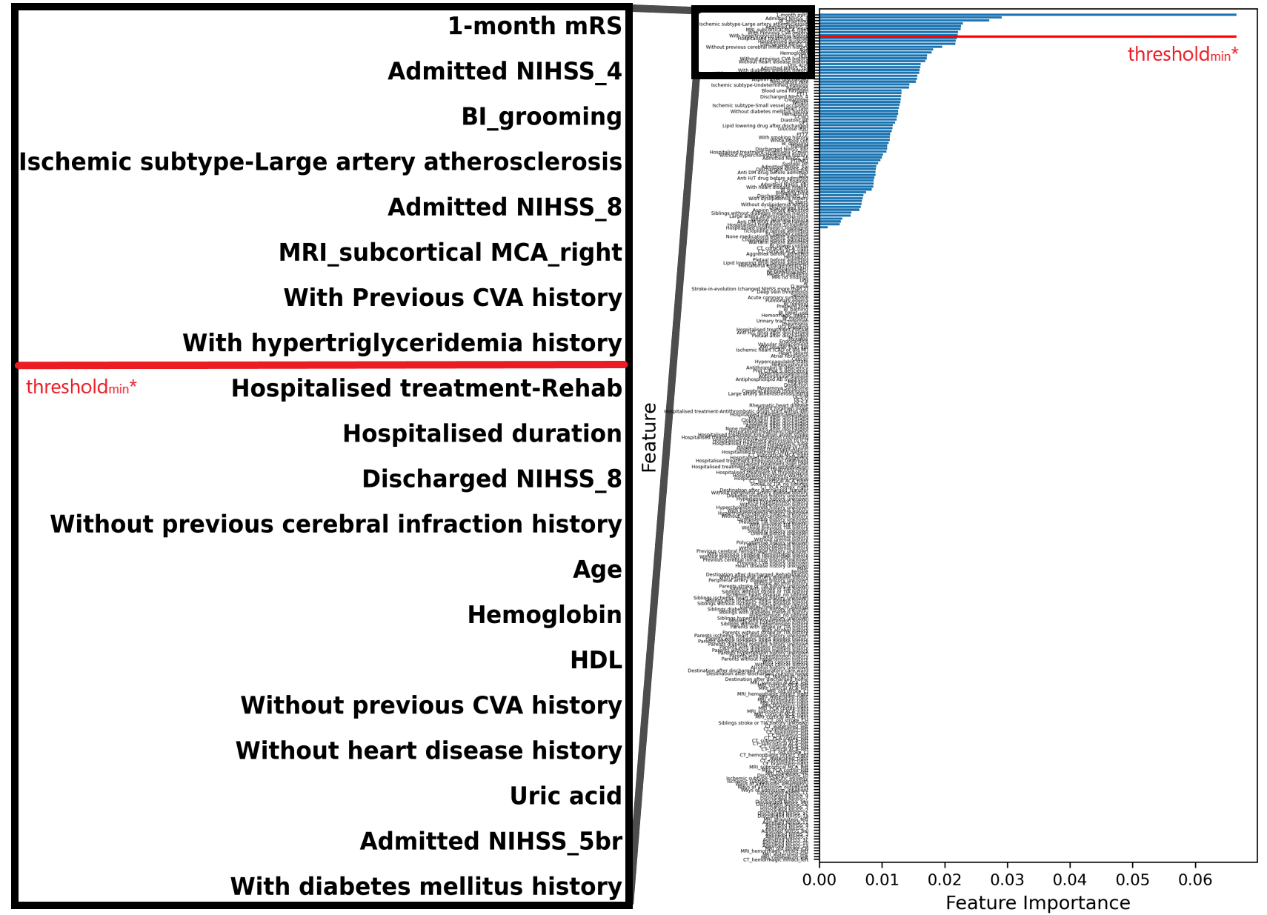


**Fig.S4.** The top 20 selected features from XGboost algorithm, which analyzed patients from good outcome at discharge to the 3-month follow-up. threshold_min_*: the number of threshold_min_’s features from good outcome at discharge to the 3-month follow-up was 8.


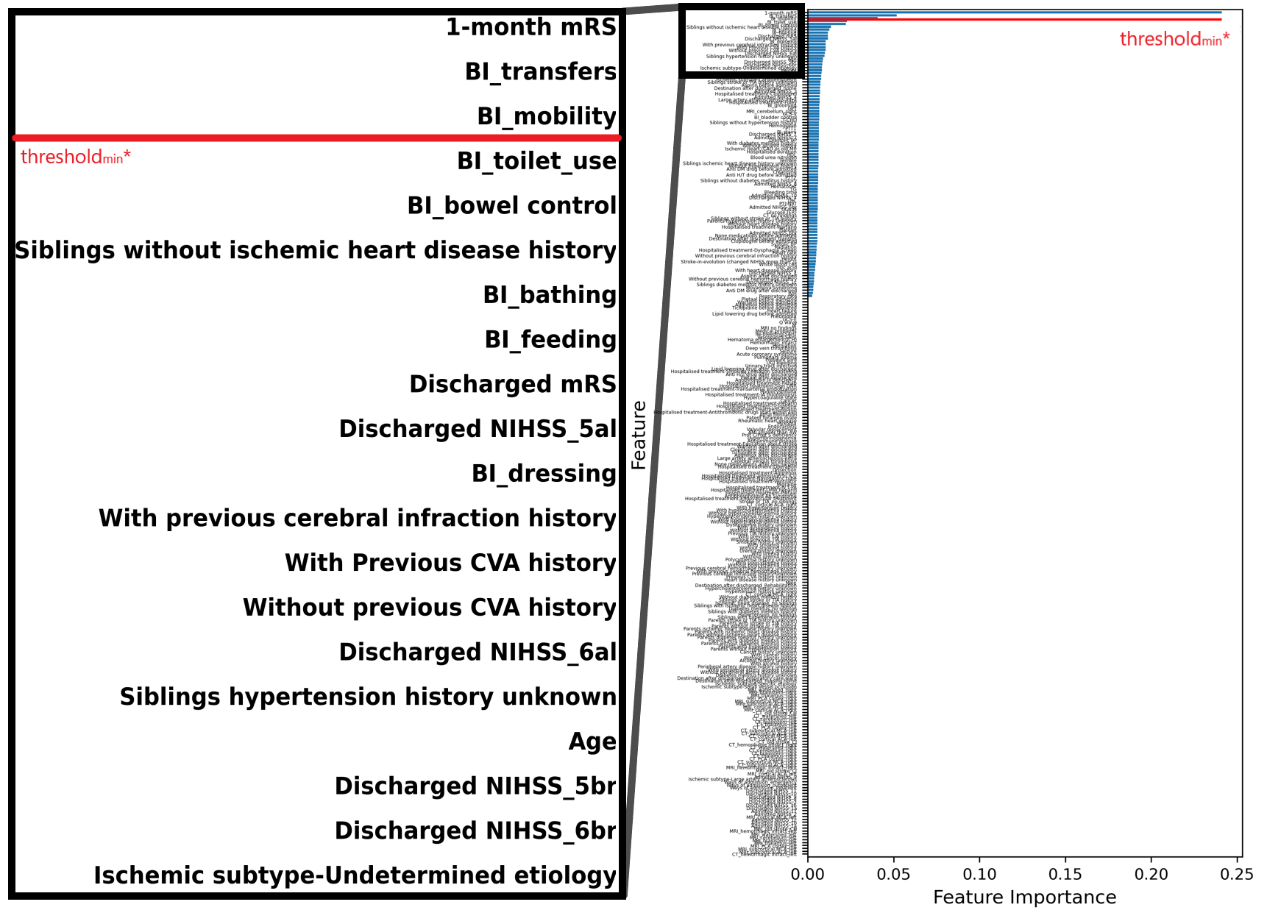


**Fig.S5.** The top 20 selected features from XGboost algorithm, which analyzed patients from poor outcome at discharge to the 3-month follow-up. threshold_min_*: the number of threshold_min_’s features from poor outcome at discharge to the 3-month follow-up was 3.

**Table S4.** Top 20 selected features of combinations of two discharge outcome groups and two prediction months. The selected features are sorted in descending order.

| **G_d_-1m** | **P_d_-1m** | **G_d_-3m** | **P_d_-3m** |
| --- | --- | --- | --- |
| BI_toilet_use | Discharged mRS | 1-month mRS | 1-month mRS |
| BI_transfers | BI_mobility | Admitted NIHSS_4 | BI_transfers |
| Discharged mRS | BI_transfers | BI_grooming | BI_mobility* |
| BI_grooming | BI_bathing | Ischemic subtype-Large artery atherosclerosis | BI_toilet_use |
| Admitted NIHSS_4 | BI_dressing | Admitted NIHSS_8 | BI_bowel control |
| BI_mobility* | BI_toilet_use | MRI_subcortical MCA_right | Siblings without ischemic heart disease history |
| Discharged NIHSS_10 | BI_stairs | With Previous CVA history | BI_bathing |
| Uric acid | BI_feeding* | With hypertriglyceridemia history* | BI_feeding |
| Hospitalised treatment-Rehab | BI_bladder control | Hospitalised treatment-Rehab | Discharged mRS |
| With hypercholesterolemia history | Hospitalised treatment-Foley | Hospitalised duration | Discharged NIHSS_5al |
| BI_stairs | Hospitalised treatment-Nasogastric tube | Discharged NIHSS_8 | BI_dressing |
| Hospitalised duration | Hospitalised duration | Without previous cerebral infraction history | With previous cerebral infraction history |
| Hemoglobin | Siblings without ischemic heart disease history | Age | With Previous CVA history |
| Without hypercholesterolemia history | BI_bowel control | Hemoglobin | Without previous CVA history |
| Height | Without previous cerebral infraction history | HDL | Discharged NIHSS_6al |
| Age | Discharged NIHSS_6br | Without previous CVA history | Siblings hypertension history unknown |
| Admitted NIHSS_10 | Hospitalised treatment-Rehab | Without heart disease history | Age |
| Ischemic subtype-Small vessel occlusion | Discharged NIHSS_10 | Uric acid | Discharged NIHSS_5br |
| Systolic BP | PTT1 | Admitted NIHSS_5br | Discharged NIHSS_6br |
| Respiratory rate | Female | With diabetes mellitus history | Ischemic subtype-Undetermined etiology |

*: the last feature of each combination’s threshold_min_; G_d_-1m: good outcome at discharge to 1-month follow-up; P_d_-1m: poor outcome at discharge to 1-month follow-up; G_d_-3m: good outcome at discharge to 3-month follow-up; P_d_-3m: poor outcome at discharge to 3-month follow-up; mRS: the modified Rankin Scale; BI: the Barthel Index; NIHSS: the National Institutes of Health Stroke Scale.

**The Receiver operating characteristic (ROC) curve of good outcome at discharge group and poor outcome at discharge group**


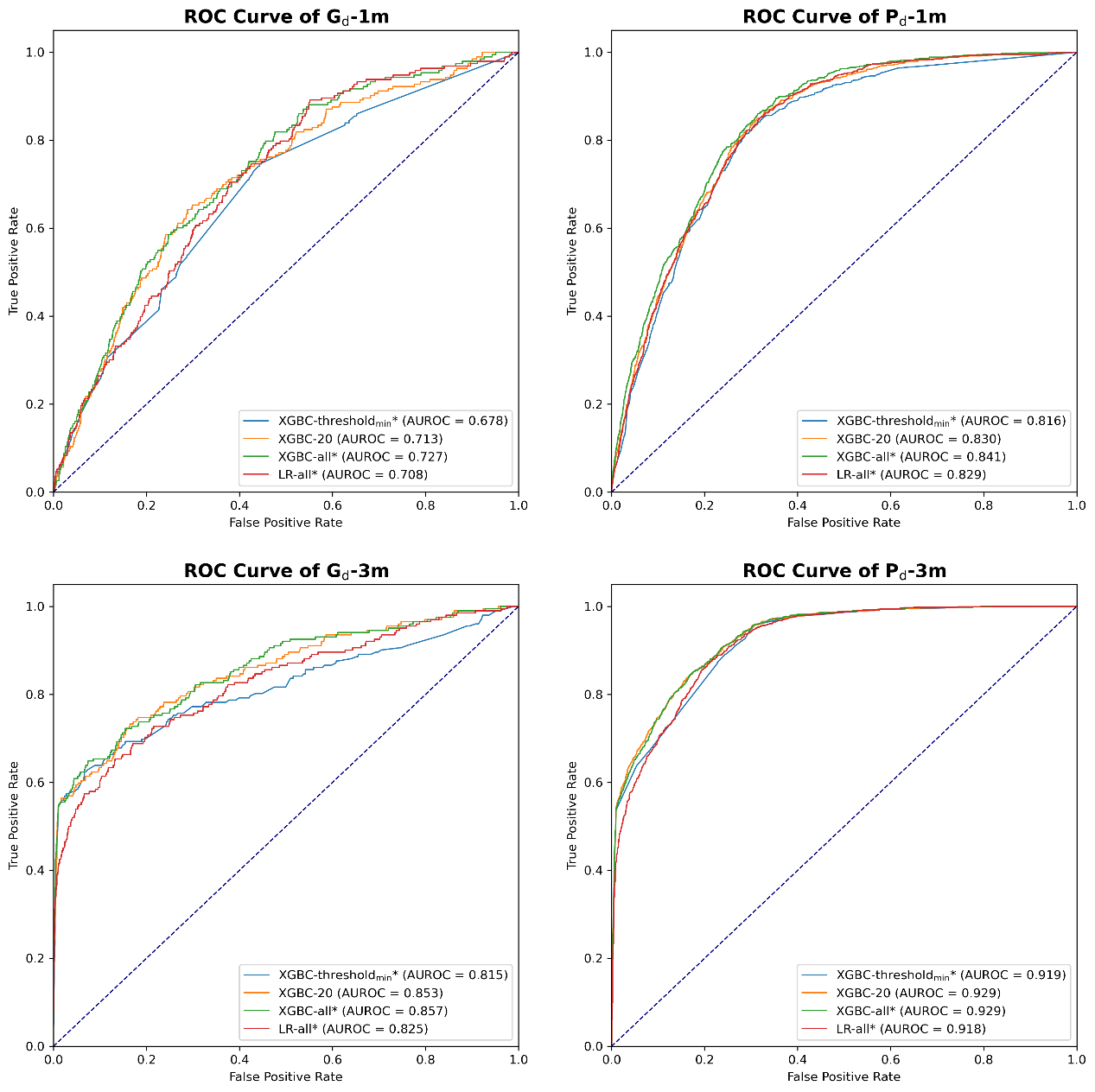


**Fig.S6.** Four receiver operating characteristic curves of combinations of two discharge outcome groups and two prediction months. G_d_-1m: good outcome at discharge to 1-month follow-up; P_d_-1m: poor outcome at discharge to 1-month follow-up; G_d_-3m: good outcome at discharge to 3-month follow-up; P_d_-3m: poor outcome at discharge to 3-month follow-up; threshold_min_*: the number of threshold_min_ of G_d_-1m, P_d_-1m, G_d_-3m, and P_d_-3m were 6, 8, 8, and 3, respectively; all*: the 1-month cohort set possessed 228 features, while the 3-month cohort set possessed 229 features.
